# Characteristic CSF Prion Seeding Efficiency in Humans with Prion Diseases

**DOI:** 10.1007/s12035-014-8709-6

**Published:** 2014-05-09

**Authors:** Maria Cramm, Matthias Schmitz, André Karch, Saima Zafar, Daniela Varges, Eva Mitrova, Bjoern Schroeder, Alex Raeber, Franziska Kuhn, Inga Zerr

**Affiliations:** 1 Department of Neurology, Clinical Dementia Center, University Medical Center Göttingen and German Center for Neurodegenerative Diseases (DZNE) – site Göttingen, Robert-Koch-Str. 40, 37075 Göttingen, Germany; 2 Slovak Medical University, Limbova 14, 833-03 Bratislava, Slovakia; 3 Prionics AG, 8952 Schlieren, Switzerland

**Keywords:** Cerebrospinal fluid, Creutzfeldt-Jakob disease, Premortem test, Prion protein, Real-time quaking-induced conversion assay

## Abstract

The development of in vitro amplification systems allows detecting femtomolar amounts of prion protein scrapie (PrP^Sc^) in human cerebrospinal fluid (CSF). We performed a CSF study to determine the effects of prion disease type, codon 129 genotype, PrP^Sc^ type, and other disease-related factors on the real-time quaking-induced conversion (RT-QuIC) response. We analyzed times to 10,000 relative fluorescence units, areas under the curve and the signal maximum of RT-QuIC response as seeding parameters of interest. Interestingly, type of prion disease (sporadic vs. genetic) and the *PRNP* mutation (E200K vs. V210I and FFI), codon 129 genotype, and PrP^Sc^ type affected RT-QuIC response. In genetic forms, type of mutation showed the strongest effect on the observed outcome variables. In sporadic CJD, MM1 patients displayed a higher RT-QuIC signal maximum compared to MV1 and VV1. Age and gender were not associated with RT-QuIC signal, but patients with a short disease course showed a higher seeding efficiency of the RT-QuIC response. This study demonstrated that PrP^Sc^ characteristics in the CSF of human prion disease patients are associated with disease subtypes and rate of decline as defined by disease duration.

**Electronic supplementary material:**

The online version of this article (doi:10.1007/s12035-014-8709-6) contains supplementary material, which is available to authorized users.

## Introduction

Prion diseases are characterized by the accumulation of abnormally misfolded prion protein (PrP^Sc^) in the brain. Spontaneous (sporadic), genetic, and transmitted forms of prion diseases are known. In sporadic Creutzfeldt-Jakob disease (sCJD), molecular characteristics of PrP^Sc^, and codon 129 genotype of the prion protein gene (*PRNP*) determine molecular sCJD subtypes [1, 2] with distinct clinicopathological phenotypes and transmission characteristics [3].

The two genetic CJDs (gCJD), examined in this study, are defined by pathogenic *PRNP* mutations of E200K and V210I. Including the mutation D178N-129M (fatal familial insomnia; FFI), these are the three most common *PRNP* mutations in Europe [4-7].

Human prion diseases are unique with respect to their self-propagating replication of the abnormally folded host-derived prion protein (PrP^C^), which in its pathological conformation (PrP^Sc^) is prone to aggregation and seeding. Various in vitro conversion assays, such as protein misfolding cyclic amplification (PMCA), enhanced quaking-induced conversion (eQuIC), or real-time QuIC (RT-QuIC) use this high aggregation and seeding activity to amplify miniscule amounts of PrP^Sc^ to a detectable level.

While this worked well with brain material in various settings, recent studies demonstrated the potential of RT-QuIC to replicate PrP^Sc^ from human cerebrospinal fluid (CSF) [8, 9]. Aggregated PrP^Sc^ detected by thioflavin-T (Th-T) can be monitored in real-time, which is a key advantage of RT-QuIC. The kinetic of the signal detection is used to evaluate the efficiency of the reaction. RT-QuIC can detect aggregated PrP^Sc^ also in various artificial prion disease models such as in the blood and the CSF of scrapie-infected hamsters [10, 11] and in scrapie-infected sheep [12].

In the present study, we applied RT-QuIC assay to human CSF and analyzed the prion seeding efficiency in humans with different forms of genetic and sporadic prion diseases. Our aim was to study the characteristics of the PrP^Sc^ seeding response in human CSF samples and not to apply the RT-QuIC as a diagnostic tool to diagnose gCJD or different sCJD subtypes. We propose that the application of the RT-QuIC method as a reliable diagnostic test for prion diseases can be extended to show that PrP^Sc^ seeds from different prion diseases convert recombinant PrP (recPrP) with a different efficiency.

## Methods

### Patients

The study included 110 prion disease patients consisting of 64 sporadic CJD, 39 genetic CJD (33 E200K-, 6 V210I mutation carriers), and 7 FFI (D178N mutation) as well as 189 control subjects. All sCJD (28 female, 36 male; aged 23–85 years; mean age 64.5 ± 1.5 years at notification; 21 MM, 25 MV, 18 VV), E200K (20 female, 13 male; aged 41–73 years; mean age 60.3 ± 1.5 years at notification, 14 MM and 19 MV), V210I (3 female, 3 male; aged 52–67 years; mean age 59.1 ± 1.9 years at notification; 5 MM and 1 VV), and FFI patients (1 female, 6 male; aged 50–85 years; mean age 62.3 ± 4.1 years at notification; 4 MM, 2 MV, 1 VV) were diagnosed as definite cases by neuropathological examinations [13–15]. Genetic evaluation for the E200K mutation was performed on blood samples derived from putative E200K carriers and the type of PrP^Sc^ was determined by autopsy.

Control group (86 female, 103 male; aged 16–87 years; mean age 64.1 ± 0.9 years at notification) composed patients with either clinically or pathologically defined alternative diagnosis. Our cohort consisted of Alzheimer’s disease patients (rapid progressive and classical forms, 35), alpha synucleinopathies (Lewy body dementia, Parkinson’s disease, 32), psychiatric disorders (psychosis, bipolar disorder, schizophrenia, 16), epilepsy (11), inflammatory and autoimmune diseases (multiple sclerosis, meningitis, 34), and other non-prion diseases (61).

The study was conducted according to the revised Declaration of Helsinki and Good Clinical Practice guidelines. Informed consent was given by all study participants or their legal next of kin.

### CSF Samples

All CSF samples were stored at −80 °C prior to analysis. The analysis of the codon 129 genotype of *PRNP* was performed after isolation of genomic DNA from blood samples according to standard methods [16].

### RT-QuIC Analysis

RT-QuIC analysis uses recPrP as a substrate to amplify small amounts of a PrP seed in human CSF to a detectable limit (comparable to a PCR for proteins). Converted and aggregated PrP can be monitored in real time by a fluorescence dye (thioflavin-T) analysis by a fluorescent reader.

Reaction buffer consisted of 162 mM phosphate buffer (pH 6.9), 170 mM sodium chloride, 1 mM ethylenediaminetetraacetic acid (Sigma-Aldrich, Munich, Germany), 10 μM thioflavin-T (Sigma-Aldrich, Munich, Germany), and 0.1 mg/ml recPrP. Eighty-five-microliter reaction buffer was seeded with 15 μl freshly thawed, neat CSF to a final volume of 100 μl. Reactions were prepared in a black 96-well, optical-bottomed plate (VWR, Hannover, Germany). Prepared plates were sealed (VWR, Hannover, Germany) and incubated in a plate reader (FLUOStar OPTIMA, BMG Labtech, Ortenberg, Germany) at 42 °C for 80 h with intermittent shaking cycles, consisting of 1 min double orbital shaking at the highest speed (600 rpm) followed by a 1-min break. Fibril formation kinetics were determined by measuring thioflavin-T fluorescent signal (450 nm excitation and 480 nm emission) every 30 min.

### Expression and Purification of Recombinant Hamster-Sheep PrP

All RT-QuIC experiments were performed using the chimera recPrP composed of the Syrian hamster residues 14 to 128 followed by sheep residues 141 to 234 of the R_154_ Q_171_ polymorph [11]. The recPrP was prepared according to the method described by Wilham et al. [17].

Briefly, DNA sequences coding for hamster (residues 90–231 and 23–231; accession K02234) and sheep (residues 25–234; accession AJ567988) recPrP residues were amplified and ligated into the pET41 vector (EMD Biosciences). Transformation of the plasmids occurs into *Escherichia coli* Rosetta cells (EMD Biosciences). RecPrP was expressed using the Overnight Express Autoinduction system (EMD Biosciences). Isolated inclusion bodies were denatured (6 M guanidine, 100 mM sodium phosphate, 10 mM tris pH 8) and bound to Ni-NTA Superflow resin (Qiagen, Hilden, Germany). Using an AKTA Explorer system (GE Healthcare), the denatured recPrP was refolded using a linear gradient to refolding buffer (100 mM sodium phosphate, 10 mM tris pH 5.8). After elution of recPrP applying a linear gradient (100 mM sodium phosphate, 10 mM Tris, 500 mM imidazole, pH 5.8), the recPrP was dialyzed against 10 mM phosphate buffer pH 5.8 overnight and filtered with a 0.2-µm syringe filter. The concentration of recPrP was determined by measuring absorbance at 280 nm. The recPrP was diluted to 0.5 mg/ml with 10 mM phosphate buffer pH 5.8 aliquoted and stored at −80 °C until analysis was performed.

As a precaution to possible batch-to-batch variability of recombinant PrP, batches were generally mixed in equal parts. Functionality of the recPrP was verified by analyzing serial dilutions of sCJD-infected brain homogenates in RT-QuIC experiments according to the method described by Wilham et al. [17].

### Determination of the PrP Concentration

To determine the PrP concentration, we used a commercial BetaPrion BSE-ELISA Test Kit (AJ Roboscreen, Leipzig, Germany) and followed the manufacturer’s instruction.

### Analysis of CSF by ELISA for 14-3-3

Levels of 14-3-3 in CSF were determined by using the CircuLex 14-3-3 Gamma ELISA Kit (BIOZOL Diagnostica Vertrieb GmbH, Eching, Germany). We followed the manufacturer’s instructions. Briefly, CSF samples were used in a dilution of 1:5 in dilution buffer. Level of 14-3-3 was detected in arbitrary units (AU) per ml (1 AU is almost equal to 1 pg). For capturing, samples were incubated for 1 h at room temperature (25 °C). After washing, 14-3-3 gamma detection antibody and later HRP conjugated anti-IgG antibody were added and incubated for 1 h at room temperature each. The colorimetric reaction was measured at 450 nm with a 1420 multilabel Counter Victor2 (Wallac) (PerkinElmer, MA, USA).

### Determination of Total Tau Level

CSF levels of total tau protein was measured using a commercially available ELISA kit (INNOTEST® hTAU Ag, Innogenetics). For the determination of tau level, we followed the manufacturer’s instructions.

Briefly, before antibody incubations, each sample (75 μl) was diluted 1:1 in sample diluent. The colorimetric reaction was measured at 450 nm with a 1420 multilabel Counter Victor2 (Wallac) (PerkinElmer, MA, USA). Each sample was measured in duplicates. For analysis, we calculated the median.

### Statistical Analyses

For investigating differences between predefined groups, three variables were defined as seeding parameters of interest. First, areas under the curve (rel. AUC, concentration over time) were calculated for each individual sample and were transformed for convenience reasons by dividing rel. AUC by the number of total measurements. Rel. AUCs were checked for being normally distributed; means (and standard deviations (SD)) of rel. AUCs were then calculated for the respective groups of interest and were compared between groups using *t*-tests and ANOVAs (+Tukey’s post-hoc tests) as appropriate. In the second step, the maximum seeding activity was calculated for each individual sample. Again, maximum values were checked for being normally distributed and then compared between group using means, *t*-test and ANOVAs (+Tukey’s post-hoc tests). In the third step, the time to 10,000 rfu was calculated for each individual sample. Times to 10,000 rfu were compared using logrank tests and Bonferroni-adjusted logrank tests (for pairwise post-hoc testing). All analyses were conducted on an exploratory basis only and were performed using SPSS 20.0 and R 2.15.

For graphical illustration, time series plots are presented at each step of the analysis displaying means (+standard errors of the mean) of seeding activity for each point in time (each measurement) in the respective groups. Thus, these illustrations show aggregated data for each time point; information about means of AUCs, individual maximums, or times to 10,000 rfu cannot be read out of these plots. Graphical “AUCs” (representing the AUC of means at each time point) do not correspond to calculated rel. AUCs (representing the mean of individual AUCs).

## Results

### RT-QuIC Assay Differentiates Between Genetic, Sporadic and FFI-Seeded Samples

**Fig. 1:**
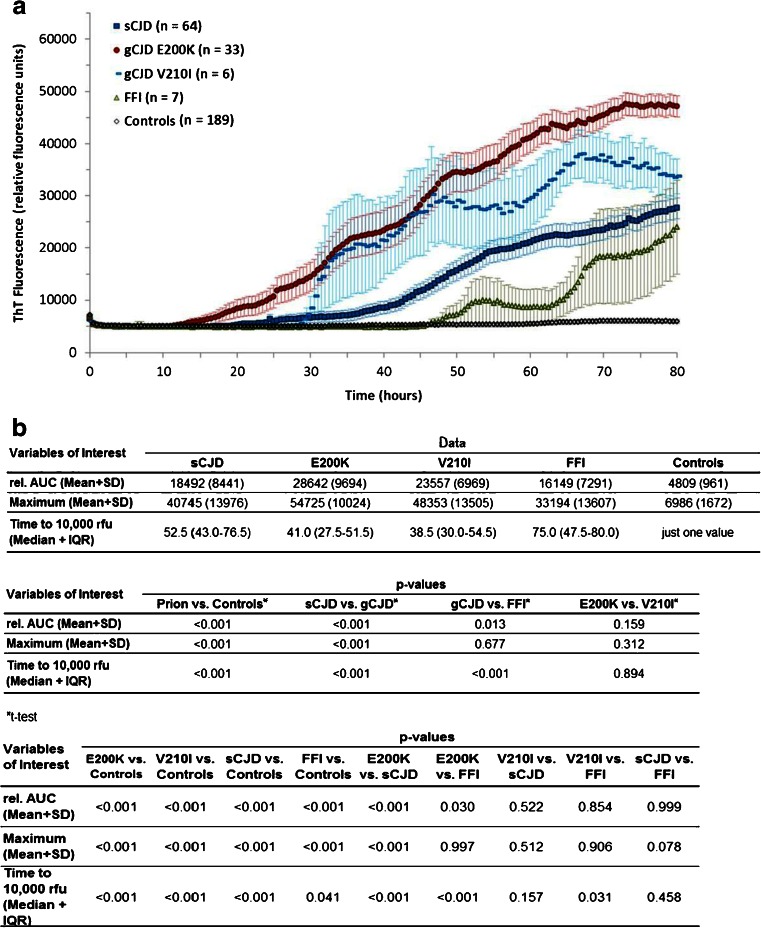
Time course of prion seeding efficiency detected by RT-QuIC in the CSF of gCJD (E200K and V210I), sCJD, FFI patients, and non-prion disease controls. **a** RT-QuIC responses of all groups were measured in relative fluorescence units (rfu) over a period of 80 h. Displayed are means + SEM (standard error of the mean) at each point in time. **b** The absolute values for rel. AUC, maximal Th-T signal (mean + standard deviation (SD)), and time to 10,000 rfu (median + IQR (interquartile range)) were shown for each group, and the *p*-values were calculated for each comparative analysis. We observed significant differences between different types of prion diseases. Genetic CJD (E200K and V210I)-seeded reactions showed the highest seeding efficiency followed by sCJD and FFI. For comparison between groups, we used ANOVAs or *t*-tests (rel. AUC) and logrank test (time to 10,000 rfu) with Tukey’s post-hoc tests and Bonferroni-adjusted logrank test as appropriate. All *p*-values < 0.05 are considered as significant

To investigate the effect of type of prion disease on RT-QuIC response, we used CSF from sCJD, gCJD (E200K and V210I), FFI, and non-prion disease control cases. The optimal running time for RT-QuIC reaction was 80 h because no additional sCJD case became positive after this time period. Therefore, a longer incubation period was not required. From each individual sample, we calculated the average from rel. AUC, times to 10,000 rfu (lag phase) and the maximal intensity of Th-T fluorescence signal over a time period of 80 h, using these parameters as indicators for the seeding efficiency of the RT-QuIC reactions (Fig. 1). The rel. AUC and the signal maximum were higher while times to 10,000 rfu were shorter in gCJD than in sCJD cases (Fig. 1a, b). The rel. AUC and the signal maximum in sCJD were not significantly different from those in FFI. The earliest signal increase to 10,000 rfu was observed in gCJD cases (median 41 h), with sCJD cases increasing significantly later (median 52.5 h). Patients with FFI had the longest lag time before the first seeding could be observed (Fig. 1a, b). The RT-QuIC response in controls was lower than 10,000 rfu.

### Impact of the Biological Characteristics of Various sCJD Subtypes

To analyze the impact of PrP^Sc^ type or *PRNP* codon 129 polymorphism on the RT-QuIC response, we compared the seeding efficiency from CSF samples derived from different sCJD patients exhibiting PrP^Sc^ type 1 or 2 (independently from codon 129 genotype) as well as different *PRNP* genotypes (MM, MV, and VV). Neither the PrP^Sc^ type nor the *PRNP* codon 129 genotype alone had a significant impact on RT-QuIC response (supplemental Fig. 1).

**Fig. 2:**
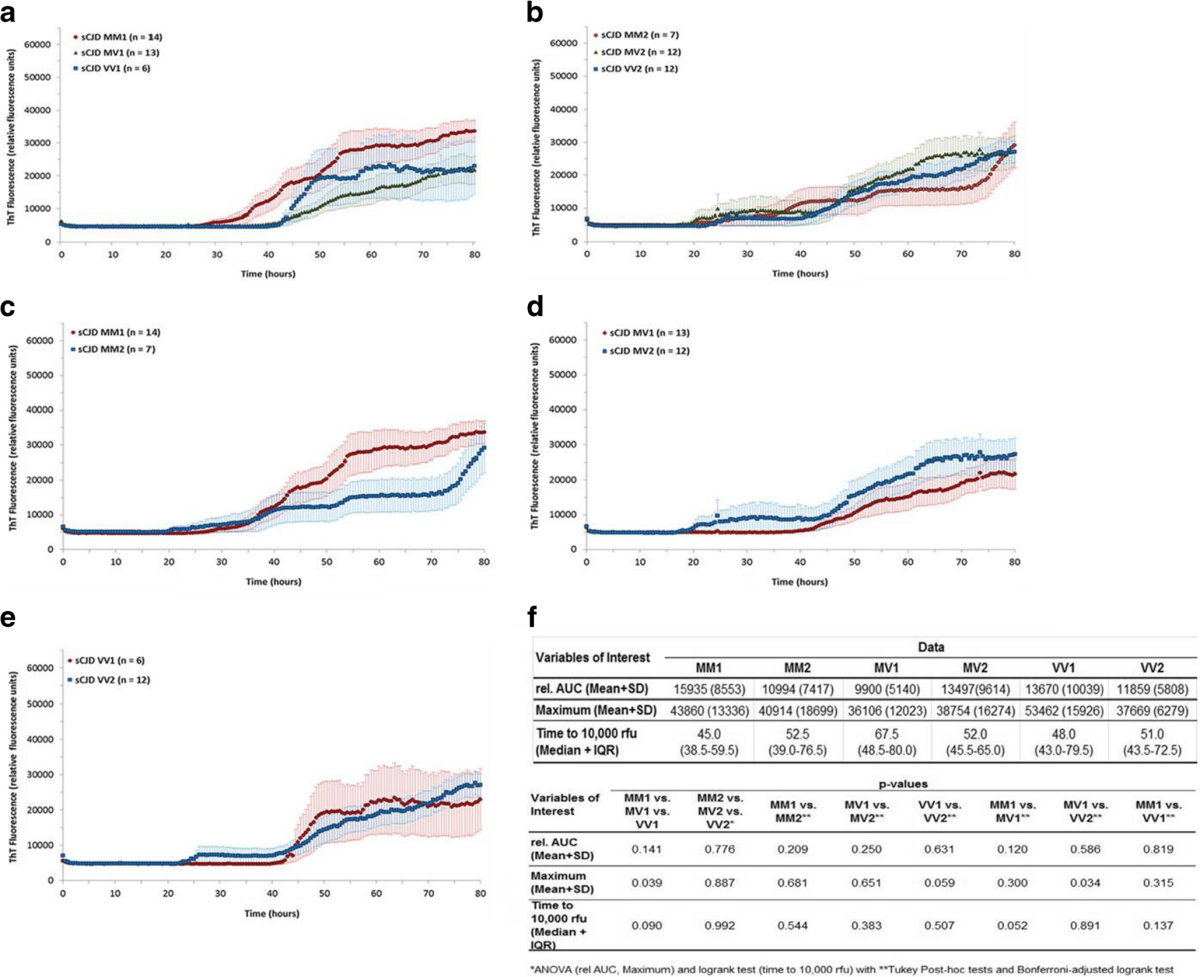
RT-QuIC analysis of the biological characteristics of various sCJD subtypes. Reactions seeded with CSF derived from sCJD patients exhibiting the same type of PrP^Sc^ with distinct *PRNP* codon 129 genotypes (MM, MV, and VV) were performed for type 1 (**a**) and type 2 (**b**). Displayed are means + SEM (standard error of the mean) at each point in time. Comparison of the RT-QuIC responses revealed a higher maximum in sCJD MM1-seeded samples compared to MV1 and VV1. (**c**–**e**) CSF-seeded reactions from sCJD patients of the same codon 129 genotype with different types of PrP^Sc^ were analyzed by RT-QuIC (mean + SEM). **f** The absolute values for rel. AUC, maximal Th-T signal (mean + SD), and time to 10,000 rfu (median + IQR) were shown for each group, and the *p*-values were calculated for each comparative analysis. For comparison between groups, we used ANOVAs or *t*-tests (rel. AUC) and logrank test (time to 10,000 rfu) with Tukey’s post-hoc tests and Bonferroni-adjusted logrank test as appropriate. All *p*-values < 0.05 are significant

We stratified the data by PrP^Sc^ type or codon 129 genotypes and analyzed the rel. AUC, the lag phase and the signal maximum of samples derived from the same type of PrP^Sc^ with distinct *PRNP* codon 129 genotypes (Fig. 2a, b). Our data revealed that the codon 129 genotype within PrP^Sc^ type 1 patients is associated with a shorter time to 10,000 rfu in MM than in MV and VV patients (Fig. 2a, f). This effect was not present in patients with PrP^Sc^ type 2 (Fig. 2b, f).

When stratifying the other way and analyzing subgroups of patients exhibiting the same codon 129 genotype but distinct PrP^Sc^ types (MM1 vs. MM2, MV1 vs. MV2 as well as VV1 vs. VV2) (Fig. 2c–e), no significant differences could be observed (Fig. 2f).

### Impact of Codon 129 Genotype in gCJD

**Fig. 3:**
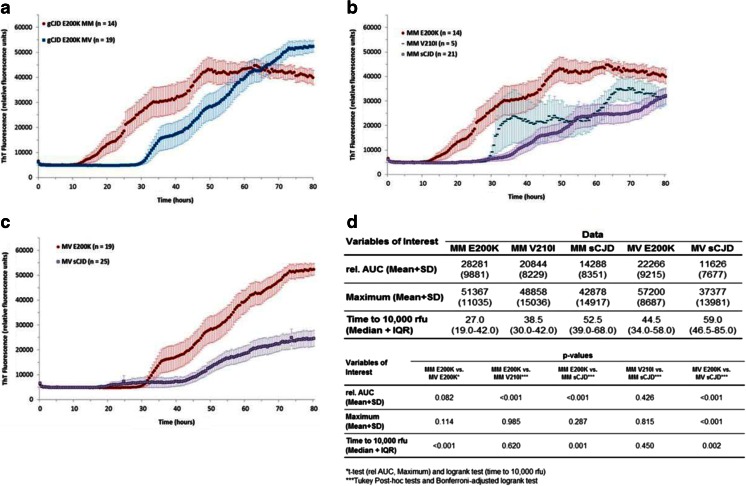
RT-QuIC analysis of different *PRNP* codon 129 genotypes in gCJD patients and different prion diseases of the same codon 129 genotype. **a** Reactions seeded with CSF derived from gCJD (E200K) patients with codon 129 genotypes MM and MV revealed a higher RT-QuIC response in MM than in MV cases. Displayed are means + SEM (standard error of the mean) at each point in time. **b**–**c** Comparison of gCJD- and sCJD-seeded reactions exhibiting either the codon 129 genotype MM or MV revealed that the type of prion disease has a significant effect on the RT-QuIC reaction (mean + SEM). **d** The absolute values for rel. AUC, maximal Th-T signal (mean + SD), and time to 10,000 rfu (median + IQR) were shown for each group, and the *p*-values were calculated for each comparative analysis. For comparison between groups, we used ANOVAs or *t*-tests (rel. AUC) and logrank test (time to 10,000 rfu) with Tukey’s post-hoc tests and Bonferroni-adjusted logrank test as appropriate. All *p*-values < 0.05 are significant

When restricting analyses to E200K patients, RT-QuIC response was significantly shorter in MM patients (median time to 10,000 rfu 27 h compared to 44 h in MV patients, *p* < 0.001), whereas there was some evidence for a difference of rel. AUC (Fig. 3a, d).

After stratifying by codon 129 genotype and restricting the analysis to MM carriers, type of *PRNP* mutation had a significant effect on the seeding efficiency in both gCJD groups (E200K and V210I) when compared to sCJD (Fig. 3b, d). Patients with E200K mutation revealed the highest seeding efficiency (median 27 h to reach 10,000 rfu), followed by V210I (median 38.5 h), sCJD (median 52.5 h) (Fig. 3b, d). This was consistent in 19 gCJD cases with MV genotype, which were compared to 25 sCJD cases from the same codon 129 genotype (Fig. 3c).

### Influence of Age at Onset, Gender, Disease Duration, and Time of Lumbar Puncture

Different disease-related characteristics and hence potential confounders were assessed for a possible association with RT-QuIC response. Age groups (younger than 60, between 60 and 70, and older than 70 years), different genders or different PrP^Sc^ strains, defined by Bishop et al. [3] showed no effect on RT-QuIC response (supplemental Fig. 1). To analyze the effect of the time from disease onset, we grouped patients in early, middle, or late disease stage.

**Fig. 4:**
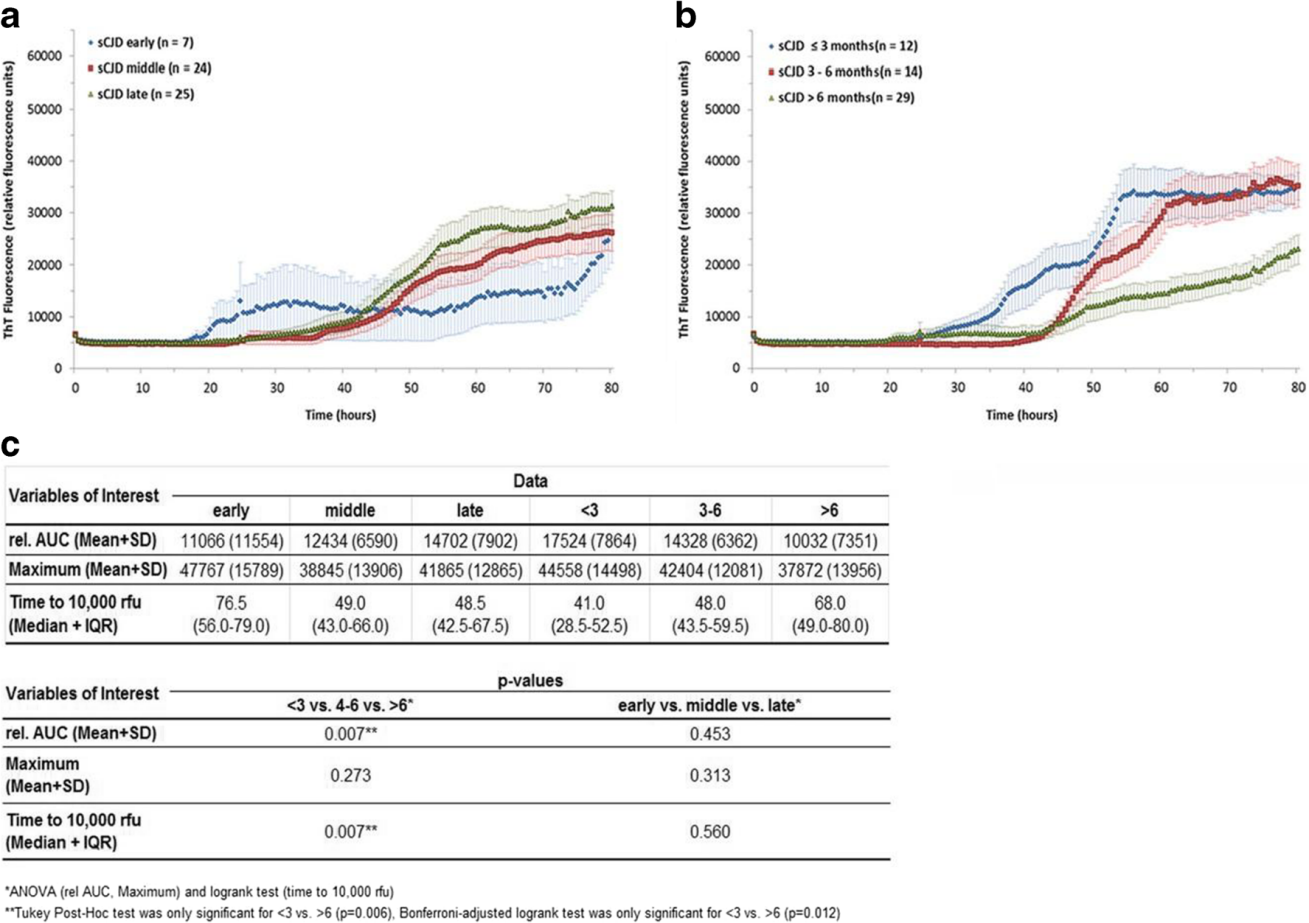
Effect of time from disease onset and disease duration. **a** RT-QuIC responses were grouped as to the time when the lumbar puncture was performed, i.e., in early, middle, or late disease stage, showing that disease stage had no significant effect on the seeding efficiency. Displayed are means + SEM (standard error of the mean) at each point in time. **b** RT-QuIC responses were grouped according to three different disease durations (<3, 4–6, and >6 months). A short duration was associated with a higher seeding efficiency. **c** The absolute values for rel. AUC, maximal Th-T signal (mean + SD), and time to 10,000 rfu (median + IQR) were shown for each group, and the *p*-values were calculated for each comparative analysis. For comparison between groups, we used ANOVAs or *t*-tests (rel. AUC) and logrank test (time to 10,000 rfu) with Tukey’s post-hoc tests and Bonferroni-adjusted logrank test as appropriate. All *p*-values < 0.05 are significant

According to Fig. 4a, sCJD (early) patients seem to have a shorter lag phase and the Th-T intensity may increase earlier compared to the other two disease stages. However, these differences are not significant because of a low number and a high variation of the RT-QuIC seeding response in this group. Unfortunately, we cannot resolve this issue because sCJD samples with an early disease state are sparely available. When analyzing the effect of disease duration, a higher seeding efficiency, indicated by a higher rel. AUC and shorter lag phase, was observed in patients with short, thus more aggressive disease followed by medium and long disease duration (Fig. 4b, c). Due to low sample numbers, stratification of the disease duration dataset by CJD subtype was not performed.

### End-Point Dilution Analysis of sCJD and gCJD Samples

**Fig. 5:**
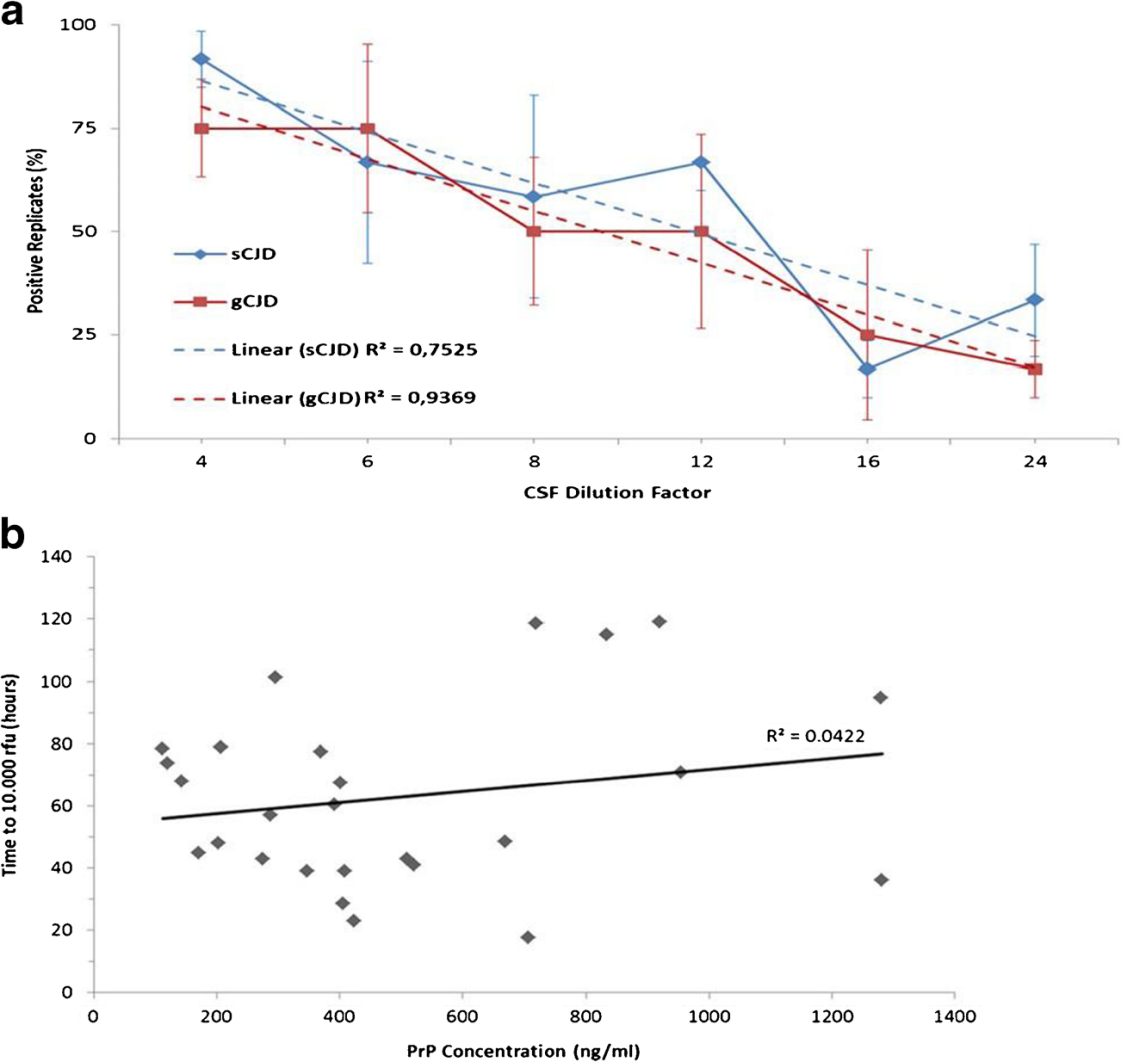
RT-QuIC end-point dilution of sCJD and gCJD CSF seeded RT-QuIC reactions. **a** CSF from sCJD (*n* = 3) and gCJD E200K (*n* = 3) was serially diluted in control CSF and used as seeds for RT-QuIC assay. We calculated percentage of positive replicates (*n* = 4). The number of positive replicates correlated with the dilution factor showing no significant differences between sCJD and gCJD patients. **b** PrP^C^ concentration in CSF was correlated to the prion seeding activity in sCJD patients. No correlation between amount of PrP^C^ and RT-QuIC response could be observed

Since gCJD exhibited a higher seeding efficiency than sCJD samples, we aimed to estimate the amount of PrP^Sc^ seed in sCJD and gCJD (E200K) CSF-seeded RT-QuIC reactions and performed an end-point analysis each with three randomly chosen samples. We used six serial dilutions from 1:4 to 1:24 and calculated the percentage of the number of positive replicates within the first 80 h for each dilution. In both groups, we observed a clear correlation between the amount of CSF seed and the number of positive replicates in the RT-QuIC assay (Fig. 5a). Sporadic CJD- and gCJD (E200K)-seeded reactions showed no significant difference in their seeding activities, which suggests an equal amount of PrP^Sc^ seeds in CSF in both groups (Fig. 5a).

### PrP Concentration is not Associated with the Seeding Efficiency

Concentration of whole PrP (which is mainly due to PrP^C^ and extremely low concentrations of PrP^Sc^) was determined by ELISA in the CSF of 26 randomly selected sCJD patients and tested for an association with seeding efficiency in the RT-QuIC assay. No association between whole PrP concentration and lag phase was observed, which indicates that the amount of whole PrP had no influence on the RT-QuIC response (Fig. 5b).

### Dependence of RT-QuIC Seeding Response from the Degree of Neuronal Damage

To investigate whether the PrP seeding efficiency depends on neuronal damage, we analyzed sCJD samples (*n* = 30) parallel by 14-3-3-, tau ELISA, and RT-QuIC. Afterwards, we correlated the RT-QuIC seeding signal response with the level of total tau and 14-3-3 protein, which are known markers of neuronal damage. However, the correlation of 14-3-3 level with RT-QuIC (real-time quaking-induced conversion) results was not significant (Spearman’s rho = 0.06, *p* = 0.74 for time to 10,000 rfu, rho=0.18, *p* = 0.19 for maximum seeding activity). The same could be observed, when we compared the tau level and the RT-QuIC seeding response (tau and time to 10,000: Spearman’s rho = 0.0486 (*p* = 0.785), tau and maximum: Spearman’s rho = −0.0848 (*p* = 0.624).

## Discussion

We studied the influence of the phenotype of human prion diseases on prion seeding efficiency in the CSF using the recently established RT-QuIC assay.

According to the data presented here, CSF from gCJD has a higher prion seeding efficiency in the RT-QuIC than CSF from sCJD and FFI patients. Only sparse information on this topic is available in the literature. Data reported by Sano et al. did not mention the difference between genetic and sporadic cases. However, looking at the individual data, differences similar to those in our study, can be seen [18].

There are several ways to explain the more rapid kinetics of the aggregation process in genetic, sporadic CJD and between different mutations in *PRNP,* such as, e.g., differences in the concentration, structure, and stability of the PrP molecule or infectivity of PrP^Sc^ (aggregates). So far, our knowledge of PrP^Sc^ characteristics in gCJD originates from studies using brain material. Accordingly, in E200K or V210I mutation carriers, the disease is caused by an altered structure of PrP^C^ [19] and the metabolism of PrP is different during life [20]. These conformational changes might engender its conversion into PrP^Sc^ by affecting the interactions with other cellular partners or the fibrillation process of PrP [21]. Also, characteristic indicators might be an increased generation of truncated fragments [20] and changed glycoform ratios of PrP^Sc^ in the brain [22].

For FFI, where the N-terminal part of PrP^C^ (23–90) plays an important role for the conversion of protein [23], several peculiar biological characteristics have been observed: low amounts of PrP^Sc^ in the brain of affected individuals, a clear effect of the codon 129 genotype on disease onset and disease phenotype, and low transmission rates in animal experiments with very long incubation times [24, 25], when compared to genetic forms of CJD (E200K) and sporadic prion diseases [4, 26, 27]. While this does not explain the early age at onset in FFI, our observation on low and slow seeding activities of FFI patients in RT-QuIC assays fits well with the known biological factors of this disease form. The molecular diversity of sporadic forms of CJD has been recognized and studied for many years and was explained at least in part by the peculiar PrP^Sc^ characteristics and *PRNP* codon 129 genotype [1, 28]. Therefore, we focused on the effect of sCJD genotype and PrP^Sc^ types on the prion seeding efficiency in the RT-QuIC assay. Patients with sCJD can be categorized according the *PRNP* codon 129 genotype and the biochemical characteristics of PrP^Sc^ into six molecular subtypes [29]. The subtypes MM1 and MV1 represent the classic sCJD phenotype and subtypes, such as MM2 or VV1, are less typical. Transmission experiments demonstrated characteristic incubation times and neuropathological lesion profiles, when brain material from patients with distinct molecular subtypes was inoculated in a panel of mice expressing different forms of human *PRNP* (MM, MV, and VV) [3]. Following these experiments and based on the transmission characteristics and PK resistances of these subtypes [30], the existence of four potential human strains was suggested: MM1 and MV1 as the classic phenotype of sCJD, MV2 and VV2, and VV1 and MM2.

Recent work using RT-QuIC assay on brain tissue demonstrated distinct RT-QuIC responses in single sCJD subtypes. In particular, atypical CJD (MM2 or VV1) or variant CJD samples showed a lower seeding efficiency compared to sCJD MM1 and MV1, indicating a delayed and slower conversion process in these samples [31].

To study the effect of the codon 129 genotype or PrP^Sc^ type in CSF separately, we performed a comparative analysis of three sCJD codon 129 genotypes (MM, MV, and VV) and two PrP^Sc^ types (1 and 2). In doing so, we tested both factors separately and examined the impact of codon 129 genotype in patient groups of the same PrP^Sc^ type. In addition, we compared sCJD patient groups with the same codon 129 genotype, but different PrP^Sc^ types, and found an effect of the *PRNP* codon 129 genotype on RT-QuIC response. Methionine homozygosity at codon 129 was associated with a higher signal maximum of RT-QuIC response, which could be shown in sCJD MM1 patients as opposed to sCJD MV1 or VV1 patients. However, we could not observe any difference when comparing the RT-QuIC responses by grouping our study participants according to the suggested four PrP^Sc^ strains [3] as defined by those experiments.

In E200K cases, the RT-QuIC assay showed a shorter lag phase and higher rel. AUC in MM than in MV genotypes, suggesting a higher seeding efficiency in homozygous patients. There is a remarkable parallel to the observation that in E200K patients, codon 129 genotype has a major influence on the risk and disease characteristics, such as earlier age at onset and shorter disease duration (3.7 +/−2.0 months compared to 7.84 +/−7.3 months) [32].

The question remains as to which factors may influence the kinetics of RT-QuIC response in addition to PrP^Sc^ type, type of *PRNP* mutation, and codon 129 genotype.

Interestingly, in sCJD patients, we found that the disease duration had an impact on the seeding efficiency of PrP, e.g., patients with short disease courses showed a higher seeding efficiency in the RT-QuIC response.

Since the whole PrP concentration has no influence on the seeding efficiency, other factors might also be important, such as the amount of PrP^Sc^ seed in the CSF. However, performing a serial dilution of gCJD and sCJD CSF samples, we could not find any differences between both groups. We suggest that conformational or structural differences in the PrP^Sc^ seed, caused by the mutation in gCJD, may be the decisive factor responsible for the increased RT-QuIC response in gCJD, rather than the amount of PrP^Sc^ seeds.

These findings together raise questions about the structure and characteristics of PrP in human CSF, which might be different from those in the brain of affected individuals. In conclusion, the data obtained confirms that the RT-QuIC assay allows us to study the characteristics of PrP^Sc^ seeding efficiency in human CSF. It clearly shows that PrP^Sc^ characteristics in the CSF of humans with a prion disease are distinct and modulated by the type of prion disease, codon 129 genotype and rate of decline as defined by disease duration. For the first time, a method can be applied to study these effects in humans affected by the disease during the symptomatic phase.

## Electronic Supplementary Material

Below is the link to the electronic supplementary material.

Supplement Fig. 1 Impact of age, gender, PrP^Sc^ strain, PrP^Sc^ type and PRNP codon 129 genotypes on the RT-QuIC response. (A) CSF samples derived from sCJD patients of different ages (younger than 60, between 60 and 70, and over 70), (B) different genders and (C) different PrP^Sc^ strains (Bishop et al., 2010), (E) different PrP^Sc^ types and (F) different PRNP codon 129 genotypes were analyzed by RT-QuIC over a period of 80 h. Comparison of the seeding activity revealed no significant influence of age, gender, PrP^Sc^ strain, PrP^Sc^ type or codon 129 genotype on the RT-QuIC response (mean + SEM). (D, G) The absolute values for rel. AUC, maximal Th-T signal (mean + SD) and time to 10,000 rfu (median + IQR) were shown for each group and the p-values were calculated for each comparative analysis. For comparison between groups, we used ANOVAs or t-tests (rel. AUC) and logrank test (time to 10,000 rfu) with Tukey Post-hoc tests and Bonferroni-adjusted logrank test as appropriate. All p-values < 0.05 are significant. (DOC 397 kb)

## Abbreviations

AUC: Areas under the curve
CSF: Cerebrospinal fluid
FFI: Fatal familial insomnia
PrP^C^: Cellular prion protein
ROC: Receiver operating Characteristic
RT-QuIC: Real-time quaking-induced conversion
rfu: Relative fluorescence units
CJD: Creutzfeldt-Jakob disease
gCJD: Genetic CJD
sCJD: Sporadic CJD
TSE: Transmissible spongiform encephalopathy
PrP^Sc^: Scrapie prion protein
