# Supplementary figures and images for: Characteristic CSF Prion Seeding Efficiency in Humans with Prion Diseases
Source: Mol Neurobiol. 2014 May 9;51(1):396–405. doi: 10.1007/s12035-014-8709-6 (PMC4309904; doi:10.1007/s12035-014-8709-6)

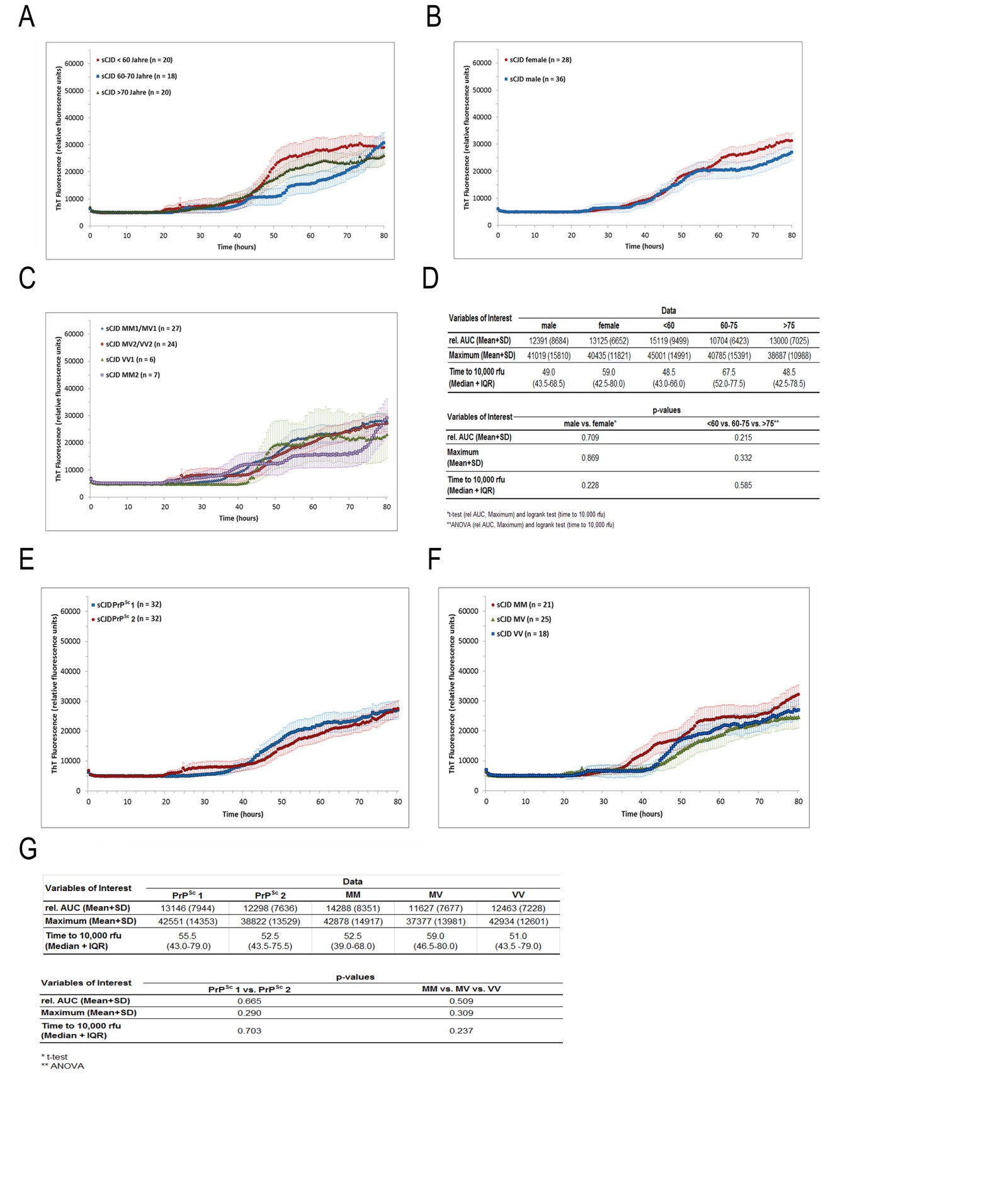

Supplement: Supplementary file 1 — Impact of age, gender, PrPSc strain, PrPSc type and PRNP codon 129 genotypes on the RT-QuIC response. (A) CSF samples derived from sCJD patients of different ages (younger than 60, between 60 and 70, and over 70), (B) different genders and (C) different PrPSc strains (Bishop et al., 2010), (E) different PrPSc types and (F) different PRNP codon 129 genotypes were analyzed by RT-QuIC over a period of 80 h. Comparison of the seeding activity revealed no significant influence of age, gender, PrPSc strain, PrPSc type or codon 129 genotype on the RT-QuIC response (mean + SEM). (D, G) The absolute values for rel. AUC, maximal Th-T signal (mean + SD) and time to 10,000 rfu (median + IQR) were shown for each group and the p-values were calculated for each comparative analysis. For comparison between groups, we used ANOVAs or t-tests (rel. AUC) and logrank test (time to 10,000 rfu) with Tukey Post-hoc tests and Bonferroni-adjusted logrank test as appropriate. All p-values < 0.05 are significant. (DOC 397 kb) [file 12035_2014_8709_MOESM1_ESM.doc]
